# The Anti-Vaccine Legacy: Re-Emergence of Subacute Sclerosing Panencephalitis in Children

**DOI:** 10.3390/neurosci7020044

**Published:** 2026-04-10

**Authors:** Maria-Delia Mihailov, Mirela Simona Manea, Ioana-Cristina Olariu, Gabriela Simona Doros

**Affiliations:** 1 Faculty of Medicine, Victor Babes University of Medicine and Pharmacy, 300041 Timisoara, Romania; olariu.cristina@umft.ro (I.-C.O.); doros.gabriela@umft.ro (G.S.D.); 2 Pediatric Intensive Care Unit, Louis Turcanu Emergency Children’s Hospital, 300011 Timisoara, Romania; 3 Department of Pediatric Neurology, Louis Turcanu Emergency Children’s Hospital, 300011 Timisoara, Romania; 4 3rd Pediatric Clinic, Louis Turcanu Emergency Children’s Hospital, 300011 Timisoara, Romania

**Keywords:** SSPE, measles, vaccine, CNS, EEG, MRI, children

## Abstract

Background: Subacute sclerosing panencephalitis (SSPE) is a chronic, progressive disease of the central nervous system (CNS) caused by persistent infection at this level with the wild measles virus. Its incidence is negatively correlated with measles vaccination coverage. The pathogenesis isn’t fully understood, but infection before the age of 2 is an important risk factor. Methods: This is a retrospective observational study conducted at the Louis Turcanu Emergency Children’s Hospital in Timisoara, Romania, based on the analysis of the medical records of patients diagnosed with SSPE between January 2021 and December 2025. We analyzed demographic and epidemiological factors, clinical and paraclinical findings, management, and outcomes. Results: Seven children were diagnosed during the study period, with a mean age of 8.4 years (range 7–11 years). Six of them had contracted measles during their first year of life, and one at the age of four. The mean latency period was 7.1 years (range 4–9 years). On admission, all patients presented symptoms consistent with clinical stage II, with periodic slow wave discharges on electroencephalogram (EEG). The initial brain Magnetic Resonance Imaging (MRI) was normal in two cases, while revealing varied abnormalities in all others. Despite complex treatment with isoprinosine and anticonvulsants, progressive cognitive and neurological deterioration continued in all patients. Conclusions: SSPE is a rare but serious, debilitating disease despite its complex, multidisciplinary care. Following a 10-year SSPE-free period, the reappearance of these pediatric cases constitutes a public health alert, unequivocally demonstrating the importance of measles vaccination.

## 1. Introduction

Subacute sclerosing panencephalitis (SSPE) or Dawson disease is a rare, yet devastating, chronic progressive CNS degenerative disease, caused by persistent infection with the measles virus at this level. The clinical onset usually occurs several years after the initial infection [1].

The name of the disease refers to its relatively rapid onset (subacute), its morphopathological changes (sclerosis), and the fact that it affects the entire brain (panencephalitis).

## 2. Epidemiology

The true incidence of SSPE is unknown, but generally, about 4 to 11/100,000 measles infections result in SSPE. In children who experienced infection within the first five years of life, this incidence is significantly higher, at 18/100,000 measles cases [2]. The risk of developing SSPE in lower- and middle-income countries has been estimated even higher, at approximately 22/100,000 measles cases [3].

Although measles elimination is an important health policy goal in many countries, recurrent outbreaks continue to occur, even in regions with robust vaccination programs. Herd immunity against measles requires vaccination of about 95% of a population, with this high level of coverage being necessary due to its extremely high contagiousness [4]. To reach this threshold, the World Health Organization (WHO) recommends that children receive two doses of the vaccine to achieve adequate coverage, prevent the spread of the measles virus, and ultimately eliminate it [5].

In the WHO European Region, between 2020 and 2022, more than 1.8 million infants were not vaccinated against measles [6]. In 2021, global coverage of the first dose of the measles vaccine was only 81%, with gradual recovery after the SARS-CoV-2 pandemic, reaching 84% in 2024—still slightly below pre-pandemic levels [7].

The WHO and the United Nations Children’s Fund (UNICEF) report that Romania’s first-dose measles vaccination coverage has dropped from 83% in 2022 to 78% in 2024. In addition, only 62% of Romanian children received the second dose in 2024 [8].

There is a compelling inverse relationship between measles vaccination and SSPE incidence. Thus, in countries where vaccination coverage is very high, the infection has been almost eradicated [9]. In addition, vaccination eliminates the risk of developing SSPE, and existing data indicate that no tissue samples taken from patients with SSPE have tested positive for vaccine virus strains [10,11].

It should be noted, however, that despite all efforts, measles cases continue to rise globally. In 2024 alone, the WHO European Region reported over 127,000 cases—the highest number since 1997, when 216,000 infections were recorded [12].

## 3. Pathogenesis

The pathogenesis of the disease is not fully understood, but it appears that the hypermutant measles virus, in combination with an abnormal cellular response and genetic susceptibility, is responsible for its onset [13].

The vaccine strain has not been associated with SSPE onset; only the wild-type virus genome has been isolated from the brain tissue of these patients [14]. To date, molecular epidemiology studies based on measles virus sequencing from the brain tissue of patients with SSPE have shown that it matches the genome circulating during the initial viral exposure [15].

Some cases of SSPE have also been reported in vaccinated children, but it has been found that most of them had been infected before vaccination, with the remaining cases attributed to rare vaccine failures [16,17,18].

After infection, the virus enters the CNS, marking a critical stage in the development of SSPE. Mutations in viral glycoproteins facilitate viral invasion of the nervous system, despite neutralizing antibodies [19]. The virus will persist and replicate in the brain, initially remaining latent, with no clinical manifestation.

Subsequently, the virus will undergo further mutations and adaptation, enabling it to continue to evade the immune system and to spread along synapses. At this stage, an inflammatory reaction occurs, leading to the destruction of neurons as well as damage to oligodendrocytes and, to a lesser extent, astrocytes, triggering the onset of the disease [20]. One proposed mechanism of neuronal dissemination involves neurokinin-1, while substance P and fusion inhibitory peptide are thought to block viral transmission [10].

In advanced stages of the disease, the inflammatory phase moves into a degenerative one, characterized by increasingly pronounced neuronal loss, demyelination, and neurofibrillary tangles [21].

From an immunological point of view, the risk of developing SSPE stems from an imbalance between overproduction of T helper 2 (Th2) cell cytokines and reduced levels of Th1 cell cytokines. This dysregulation results in impaired humoral immunity and deficient cytotoxic responses, enabling viral replication and persistence within neuronal cells [22].

Genetic susceptibility of the patient is a significant contributing factor in the development of SSPE, with parental consanguinity also being reported [23,24].

An important risk factor is measles infection during the first two years of life, as the immaturity of cellular immunity at this early age is believed to play a key role in the higher incidence of SSPE [25]. This early infection is associated with a shorter latency period before SSPE becomes clinically apparent [26]. Other risk factors include low socioeconomic and educational status, large families with many children (which increases the likelihood of sibling transmission), and newborns whose mother contracted measles during pregnancy [27].

The WHO has mentioned vitamin A deficiency as a risk factor for measles infection, given the contribution of this vitamin in maintaining epithelial integrity and immune system function, but its role in the pathogenesis of SSPE has not been fully established [28,29].

## 4. Diagnosis

### 4.1. Clinical Features

The onset of the disease typically occurs 7–11 years after a primary measles infection, though some individuals develop SSPE symptoms within the first year post-infection [30]. while others, even after a latency period of decades [31,32,33].

Some cases can experience an atypical onset with psychiatric manifestations (such as schizophrenia, catatonia, atypical psychosis) and gait disorders, isolated extrapyramidal signs, or optic atrophy, without following the normal course of the disease [34].

The diagnosis remains challenging due to this prolonged latency period between measles infection and SSPE onset. Symptoms typically begin insidiously and progress subacutely, manifesting as cognitive decline, behavioral and personality changes, and progressive motor deterioration. Subclinical symptomatology is more common at a younger age [35].

As the disease advances, paroxysmal movements, spasms, periodic myoclonic jerks, dystonia, tremor, chorea, rigidity, pyramidal and extrapyramidal signs, and ataxia appear, causing gait disturbances and falls from a standing position [36]. While myoclonic jerks don’t impair awareness, seizures typically involve loss of consciousness, particularly tonic–clonic episodes, and can sometimes be difficult to treat [37]. Focal or atonic seizures can also pose significant therapeutic challenges [38].

After months or years of progression, coma sets in, patients become akinetic and mute, and ultimately enter a vegetative state [39].

There is a staging of the clinical course (Table 1), though it is important to note that motor regression occurs in all cases [40]. In general, the progression from stage I to the final stage of the disease takes between 1 and 5 years, followed by death [41,42].

### 4.2. Laboratory Investigations

#### 4.2.1. Cerebrospinal Fluid (CSF) Examination

Since the measles virus is confined intracellularly in the CNS, the polymerase chain reaction (PCR) for measles is negative. Determination of immunoglobulin G (IgG) anti-measles antibodies in CSF by the enzyme-linked immunosorbent assay (ELISA) is crucial for diagnosing SSPE, as this method has 100% sensitivity and over 90% specificity [43].

#### 4.2.2. EEG

EEG is a valuable diagnostic method. It can detect pathognomonic changes consisting of slow, high-amplitude biphasic waves that appear bilaterally, symmetrically, and synchronously at fixed and regular intervals, called slow wave complexes or Radermecker complexes, which usually appear after at least four months of disease progression [44,45]. In addition, generalized tonic–clonic and tonic seizures or focal and multifocal epileptiform discharges can be documented [46].

#### 4.2.3. Brain MRI

Brain MRI can provide important information, but it is not an essential diagnostic method and does not correlate with the clinical stage of the disease [47]. Moreover, it may appear normal in the early stages or reveal only mild abnormalities in the white matter, but it is useful for disease progression monitoring. As the condition advances, the investigation may reveal a decrease in gray matter volume, periventricular demyelination, and cerebral atrophy, leading to marked ventriculomegaly [48].

#### 4.2.4. Magnetic Resonance Spectroscopy

Magnetic resonance spectroscopy (MRS) offers insights into in vivo brain metabolism and neuronal function, enabling early disease detection [49]. The findings can reveal decreased N-acetyl aspartate due to neuronal loss, increased choline from demyelination and inflammation, increased myo-inositol from active gliosis, and elevated lactate from macrophagic infiltration [50].

#### 4.2.5. Brain Tissue Biopsy

Brain tissue biopsy reveals inflammatory changes and neural loss and detects measles virus antigens or viral RNA. It is rarely indicated, only when the other mentioned test results are inconclusive [51].

To diagnose the disease, clinical suspicion is crucial, supported by immunological evidence, namely an increase in anti-measles virus antibody titer in the CSF/serum. Diagnosis requires two major and one minor criterion according to Dyken’s criteria (Table 2) [52]. If the presentation is atypical, brain biopsy and/or molecular diagnostic tests may be needed.

## 5. Treatment

Currently, there is no effective curative treatment, and the disease progresses towards death in the majority of cases, despite the use of immunomodulatory or antiviral therapies.

Isoprinosine (methisoprinol) is the most commonly used drug in the treatment of SSPE due to its immunomodulatory effect, activating immune cells, lymphocyte proliferation, cytokine production, and enhancing natural killer cell cytotoxicity [53,54]. The recommended dose is 50–100 mg/kg/day, with treatment lasting from several months to 2–3 years or, in some cases, for life. According to some studies, it can slow the progression of SSPE, but its certain beneficial effect has not yet been proven [55,56,57].

Interferon alpha (IFN-α) modulates the immune response, inhibits viral replication, and stimulates antiviral activity. It has been used as monotherapy or in combination with oral isoprinosine or intravenous ribavirin [58,59,60].

IFN-α is administered mostly by intrathecal route, as direct delivery into the CNS maximizes its antiviral effects. The standard dosage ranges from 1 to 3 million international units (IU) administered two or three times weekly. For this reason, some medical centers are using Ommaya reservoirs to simplify the frequent administration process [61,62]. Recently, a surgical technique has been described involving the placement of an intraventricular catheter connected to a rechargeable subcutaneous pump. This pump was refilled with 9,000,000 I.U. of α-IFN approximately every 21 days, enabling continuous administration with a constant cerebral concentration and a lower medication toxicity [63].

The intravenous route could also be an option, but it may lead to more pronounced systemic side effects. The dosage is 3–10 million IU/m^2^ of IFN-α three times a week [64].

Interferon beta (IFN-β) has been used considerably less in SSPE treatment, compared to IFN-α. A retrospective study that compared two different regimens of IFN-β: 60 mcg intramuscular once weekly or 22 mcg subcutaneously thrice weekly, both combined with oral isoprinosine 50–100 mg/kg per day, found that the thrice weekly regimen may be an effective treatment option in SSPE [65].

Immune therapy with rituximab, intravenous immunoglobulins, or corticosteroids, as well as plasma exchange were used without any evidence of long-term benefit [13].

Amantadine, an antiviral agent with mild antiparkinsonian activity, has gained attention in recent years due to its presumed immunomodulatory effect, but its administration has not led to any convincing results [66].

Other therapeutic approaches, still under investigation, showing some promising results, include nucleic acid analogs with antiviral effects like ribavirin, favipiravir, and remdesivir. These have demonstrated partial symptom relief or slowed disease progression in certain patients [67,68]. An evident clinical improvement was obtained in patients who started intraventricular ribavirin treatment in the early stages, underlining the importance of early diagnosis and treatment of the disease [69].

It is important to highlight the major role of a comprehensive, multidisciplinary therapeutic plan, with emphasis on symptom management, supportive measures, and palliative care.

## 6. Evolution

As mentioned above, there is currently no effective treatment, so that in most cases, death occurs within 3–4 years after onset [70]. Fulminant evolution was also described, where the progression to akinetic mute stage or to death was reached within 6 months from the onset of symptoms, probably due to genetic predisposition [71]. In stage IV, patients usually die from sepsis, severe bulbar involvement, or hypothalamic instability [72].

The mortality rate is extremely high (approximately 95%), but there are some reports of spontaneous regression or unusually long survival [73,74,75,76,77].

## 7. Case Reports

This is an observational retrospective study conducted at the Louis Turcanu Emergency Children’s Hospital in Timisoara, Romania, based on an analysis of the medical records of all patients admitted and diagnosed with SSPE between January 2021 and December 2025.

We present seven consecutive patients with SSPE admitted, diagnosed, treated, and followed during the study period. One patient was diagnosed in 2021, two in 2022, one in 2023, two in 2024, and one in 2025.

We aimed to analyze demographic data (age and sex distribution), epidemiology (measles infection and vaccination status), clinical data (latency period, symptomatology on onset, clinical presentation at hospital admission, and clinical stage on admission), paraclinical diagnostic, treatment, and clinical outcome.

### 7.1. Demographic Data

The sex distribution revealed a male preponderance, with 5 males (71.4%) and 2 females (28.6%). The patients’ age at diagnosis ranged from 7 to 11 years, with a mean age of 8.4 years (SD = 1.2).

### 7.2. Epidemiologic Data

All children in our cohort had measles infection, with 6 cases occurring during their first year of life (the earliest at 1 month old) and one patient at 4 years old. The measles vaccine was administered only to three children who had the infection in their first year of life, two of whom received a single dose at the age of 1 year, while the other received both doses at 1 and 5 years of age. The child who contracted measles at the age of 4 years had not been vaccinated at all.

### 7.3. Diagnosis

#### 7.3.1. Clinical Features

The onset of the disease occurred after a mean latency period of 7.1 years (SD = 1.5) following measles infection, ranging from 4 to 9 years. Up to that point, all children had normal age-appropriate neuropsychological development, with no pre-existing chronic conditions or immunosuppression.

It is interesting to point out that the first signs of illness occurred between 2 weeks and 14 months before hospital admission, the mean period being 3.9 months (SD = 4.6). All patients were referred to our hospital due to a sudden worsening of the neurological condition.

Case 1.

The first patient is a 9-year-old girl, known to have contracted measles at the age of 1 year, without subsequent vaccination, who was admitted to our hospital in 2021, one month after the first clinical signs of the disease, which consisted of insomnia, strange behavior, visual hallucinations, and psychomotor agitation.

Upon admission, she remained conscious but presented repetitive periodic myoclonic jerks at a regular frequency of about 10 s in the upper and lower limbs, accompanied by lower limb weakness, frequent stumbling, gait and balance disturbances, and swallowing difficulties.

Case 2.

The second case involves an 11-year-old boy who was transferred to our hospital in 2022 from a regional hospital. The patient had contracted measles during his first year of life, at 9 months of age, requiring hospitalization. He was not subsequently vaccinated.

Initial symptoms appeared 14 months before admission and consisted of memory impairment, regression in acquisition skills, and intentional tremor in the upper limbs. He was diagnosed with attention deficit hyperactivity disorder (ADHD), and treatment with atomoxetine was initiated.

Subsequently, his memory impairment worsened, he lost the ability to write or perform calculations, developed eye deviation, myoclonic jerks in both upper and lower limbs, and an unsteady gait leading to frequent falls.

At the time of admission to our hospital, his consciousness was preserved, but he presented, in addition to the above symptoms, decreased muscle strength, a positive Babinski sign, and could walk only short distances with instability.

Case 3.

The third case involved an 8-year-old boy who was admitted to our hospital in 2022. He had measles at 8 months of age and was not subsequently vaccinated.

The symptoms began 3 months before admission, with cognitive regression. Gradually, he developed gait and balance disturbances, myoclonic jerks in his upper limbs, followed by generalized tonic–clonic seizures, requiring hospital admission.

Case 4.

The fourth case involves a 7-year-old male patient who presented at our hospital in 2023 with motor regression, tetraparetic motor deficit, extrapyramidal movements, myoclonic jerks in both upper and lower limbs, and a complete absence of expressive language.

At six months old, he experienced a measles infection complicated by pneumonia and febrile seizures. He was treated with valproic acid, after which no further seizures occurred, and his psychomotor development progressed normally. He was further vaccinated at 1 year of age.

Symptoms emerged one month before admission, starting with fatigue, sialorrhea, speech disorders, and walking difficulties. As the condition progressed, the parents described episodes in which the child seemed unwilling to speak, along with symptoms that could be interpreted as absence seizures.

Upon admission, the patient was conscious but exhibited comprehension deficits, being able to execute only simple commands. Speech was limited to a few words. He also presented extrapyramidal signs, myoclonic jerks in the upper and lower limbs, and stereotypical movements.

Case 5.

The fifth patient is an 8-year-old girl, diagnosed with SSPE in 2024. It should be noted that she had measles at ten months of age and subsequently received only a single dose of vaccine at the age of 1.

Symptoms began two weeks before presentation in our clinic and consisted of temporal-spatial disorientation, mixed insomnia, behavioral disorders, unmotivated situational scratching, sialorrhea, bradylalia, aphasia, and myoclonic jerks in the upper limbs, as well as difficulties in maintaining the upright posture and walking. As these symptoms progressed, she experienced a generalized tonic–clonic seizure, requiring hospitalization.

At admission, the patient was confused, with poor expressive language and an inability to understand verbal commands. She also presented severe dysphagia, myoclonic jerks on the upper and lower limbs, an unsteady gait, and was able to walk only short distances.

Case 6.

The sixth patient is an 8-year-old boy who had measles infection at one month and was subsequently vaccinated at 1 and 5 years of age. He was admitted to our hospital, where he was diagnosed with SSPE in 2024.

It should be noted that, 4 months before admission, he experienced generalized tonic–clonic seizures and was treated with carbamazepine. Following this episode, progressive cognitive regression occurred, with loss of both receptive and expressive language skills.

Two months later, he suffered a new generalized tonic–clonic seizure, leading to hospitalization, where carbamazepine was replaced with valproic acid.

Under this treatment, myoclonic seizures occurred, accompanied by loss of muscle tone, leading to posterior head drops. He subsequently developed echolalia, and his cognitive regression worsened further, manifesting as poor attention, inability to recall object names, and temporal-spatial disorientation. His motor decline also progressed steadily.

Upon admission to our hospital, in addition to the previously mentioned symptoms, he also exhibited a complete absence of expressive and receptive language, extrapyramidal rigidity, and an inability to sit, stand, or walk. Active movements were limited to the upper and lower limbs, and severe dysphagia was observed.

Case 7.

The seventh case involves an 8-year-old male patient who has not been vaccinated and was diagnosed with measles at the age of 4 years.

Four months before this admission, he presented with atonic seizures requiring valproic acid treatment. Under this medication, he developed apathy, language regression, and upper limb tremors.

He was admitted to our hospital in January 2025 due to disease progression, which was marked by significant sialorrhea, swallowing difficulties, and repetitive myoclonic jerks on the right side of the body. Orthostasis and walking were possible only with support.

At the time of diagnosis, the patients were already in clinical stage II (2 in stage IIA, 3 in IIB, and 2 in IIC). Clinical presentation was characterized by a wide spectrum of symptoms, though all cases exhibited a suggestive neurological profile.

The diagnosis was established based on Dyken’s criteria. Clinical suspicion played a key role, confirmed by further investigations: IgG anti-measles in serum and CSF (determined by ELISA), EEG, and MRI.

#### 7.3.2. Paraclinical Diagnostic

Autoimmune pathology, immunodeficiency, and other viral infectious etiologies were excluded in all patients.

**Figure 1:**
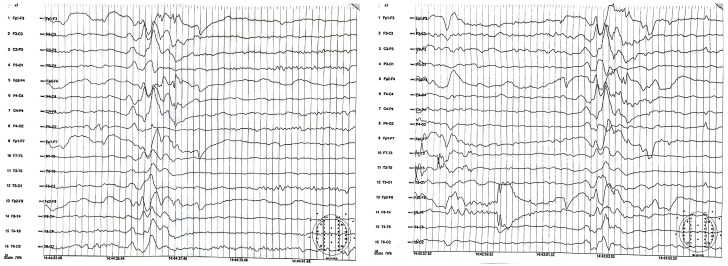
EEG of case 5: Slow background electrical activity with bilaterally synchronous high-amplitude slow wave complexes, recurring at regular intervals.

**Figure 2:**
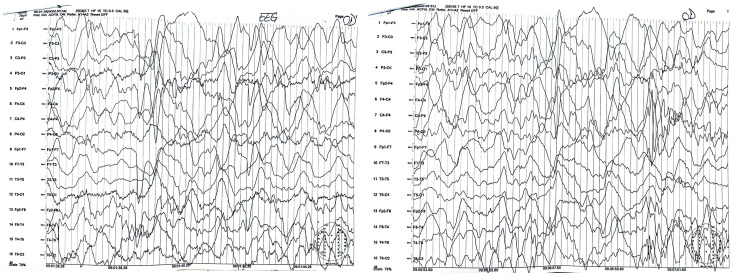
EEG of case 6: Diffuse slow background electric activity with generalized rhythmic synchronous discharges of slow wave complexes and spikes occurring at relatively regular intervals.

**Figure 3:**
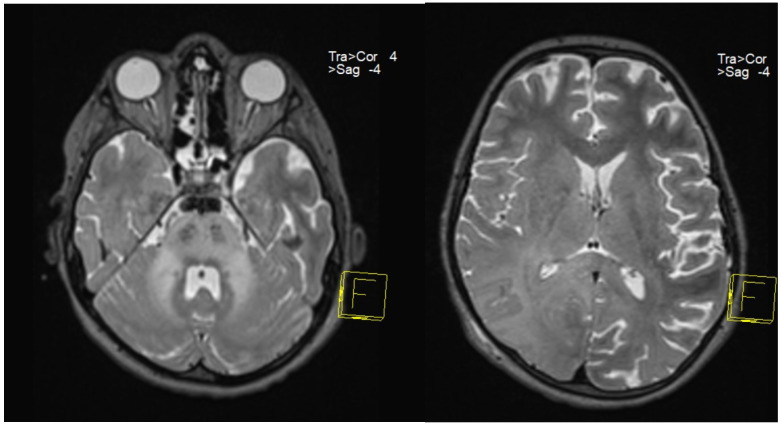
MRI performed on case 6: Symmetrical generalized T2 hyperintensity at the pontine level and middle cerebellar peduncles, with additional involvement in the supratentorial temporo-parieto-occipital regions and right frontal cortico-subcortical areas, in the corpus callosum and left parieto-occipital region.

Following clinical suspicion, to confirm a positive diagnosis of SSPE according to Dyken’s criteria, patients underwent EEG (Figure 1 and Figure 2), lumbar puncture with anti-measles IgG titer measurement in CSF (along with anti-measles IgG serum titer determination), and MRI (Figure 3).

All patients in our study group had high titers of measles IgG antibodies in both serum and CSF, demonstrating clear evidence of intrathecal IgG anti-measles antibody production. EEG showed periodic generalized bilaterally synchronous slow waves in all cases, while MRI findings varied significantly, being normal in two patients and showing moderate to extensive abnormalities in the other five (Table 3). Since all patients met Dyken’s criteria, a brain biopsy was not required to confirm the diagnosis.

### 7.4. Treatment

Given the patients’ severe and complex clinical symptoms, they received comprehensive multidisciplinary medical care. It should be noted that due to the broad neurological presentation upon admission, all patients were initially treated with intravenous dexamethasone. However, once SSPE was diagnosed, this treatment was tapered and discontinued.

Since there is no consensus for a therapeutic strategy and no treatment guidelines are available, oral isoprinosine at a standard dose of 100 mg/kg/day was our option for all patients after SSPE criteria were fulfilled. This drug was chosen because it has shown some beneficial outcomes, having the same function as Interferons, while being easy to administer, avoiding the complications associated with intrathecal IFN administration [78].

Only one patient (case 4) received combined therapy with amantadine for 5 months. This therapeutic regimen was indicated and initiated outside the country, without clinical improvement, so it was discontinued, and isoprinosine was continued as monotherapy for one year, after which, due to disease progression to stage IV, it was also discontinued.

All patients required combined anticonvulsant therapy, with a third drug needed in three cases (Table 4). Levetiracetam was used in all patients for myoclonus management, in association with valproic acid (3 cases), valproic acid and clonazepam (2 cases), valproic acid and carbamazepine (1 case), and carbamazepine and clonazepam (1 case).

### 7.5. Clinical Outcome and Follow-Up

All patients were monitored, treated, and followed up in our hospital by a multidisciplinary team. At the end of the study period, only five of the patients remained under our monitoring and treatment.

Case 1 is currently in stage IV and receives medical care in a rehabilitation center outside the country, where his family moved six months after the initial diagnosis, when the patient was in stage III.

The patient representing case 7 was referred to a rehabilitation center 4 months after a SPPE diagnosis was made, where he died from respiratory complications.

Consistent with previously reported data, the combination therapy of levetiracetam and valproic acid resulted in a slight decrease in seizure activity in all patients [79]. Carbamazepine has been shown to have a beneficial effect on myoclonus, which has also been observed in our patients [58].

It should be noted that after initial admission, despite rapid initiation of treatment, once the diagnosis was established, the clinical condition continued to worsen, with all patients progressing to stage III of the disease within the subsequent months. Their clinical evolution led to fulfilling the criteria for admission to the intensive care unit, requiring their transfer to this unit, where treatment and monitoring continued.

Swallowing difficulties have required a temporary nasogastric tube for short-term enteral nutrition, followed by a permanent gastrostomy as symptoms progressed. Currently, the three patients in stage III can breathe spontaneously, while stage IV of the disease requires mechanical ventilation via the tracheostomy tube.

All five patients are currently receiving home care and are being regularly monitored at our hospital. After an average follow-up period of 26.2 months (range: 2–41 months), no clinical improvement was observed in any patient. Three of them showed relative stabilization of the disease at stage III, while the other two progressed to stage IV (Table 4).

In patients whose disease had stabilized at stage III, we opted to continue isoprinosine administration, whereas in those exhibiting clinical deterioration, it was discontinued.

## 8. Discussions

This recent resurgence of SSPE cases, with 1–2 new cases being diagnosed annually in our hospital, has drawn our attention and raised significant concerns, especially since no child was diagnosed with this disease in our institution in the decade preceding the SARS-CoV-2 pandemic.

All children in our series had measles infection in the first years of life, six of them in the first year and one at the age of four, which explains the early onset and also the severe disease progression, poorly influenced by the treatment. An evident male preponderance (71.4%) was observed. The patients’ age at diagnosis ranged from 7 to 11 years, with a mean age of 8.4 years (SD = 1.2).

The initial clinical presentation varied widely, but unfortunately, we had no stage I cases, and all seven patients presented in stage II. Following diagnosis, despite treatment initiation, our patients experienced a rapid clinical deterioration progressing to stage III within the next few months. It is possible that the initial administration of dexamethasone, before the diagnosis of SSPE was made, may have contributed to the rapid progression of symptoms, because steroid therapy has previously been associated with fulminant progression of SSPE [22].

Of the seven patients, one died approximately five months after diagnosis in a rehabilitation center, and one patient is currently being treated at a rehabilitation clinic outside the country. It is difficult to conclude whether the relative stabilization observed in the three patients who are currently in clinical stage III is due to the therapy or is actually a result of the natural progression of the disease, which is known to vary from case to case. Unfortunately, no clinical improvement was achieved in any case; two of the five children who are currently under our monitoring and follow-up reached the terminal stage of the disease.

Our series of seven cases of SSPE occurred over 5 years in previously healthy children, with a catastrophic neurological deterioration, is alarming and demands immediate action from the health authorities.

This resurgence of SSPE has also been reported recently in other European countries, highlighting the need for a collective effort to eradicate this serious complication of measles infection [9,80].

In recent years, particularly due to the pandemic, childhood vaccination programs, including measles immunization, have suffered global disruptions. In addition, there is a growing tendency among parents to refuse to vaccinate their children [81].

In Romania, the current vaccination schedule recommends the administration of the first dose of measles vaccine at 1 year of age and the second dose at 5 years of age. This schedule leaves the most susceptible children, whose natural immunity is the weakest, without protection, because it has been proven that the young age at which a child contracts measles is a risk factor for the subsequent development of SSPE, and children under one year of age have a risk of contracting measles.

Measles exposure before protection offered by vaccination highlights the crucial role of herd immunity in protecting infants from infection. Unfortunately, these measles outbreaks are being observed in more and more countries, including those with well-established vaccination programs [82].

A recent report from the European Union shows that, between 1 November 2024 and 31 October 2025, 30 EU/EEA Member States recorded 9603 cases of measles, 6868 (71.5%) being laboratory confirmed. Romania reported the highest number of cases at 5994 [83].

Early diagnosis of this severe disease is crucial because it has been found that patients diagnosed with clinical stage I or II before treatment tend to have higher success rates. Another observed pattern is that all treatments remain effective only for a limited duration before losing efficacy, even when dosages are increased [83]. However, all current therapeutic strategies define treatment as successful if it leads to symptom improvement, slowing of disease progression, and increased survival, as a complete cure is not possible.

Given that the individual genetic profile may confer a higher predisposition to SSPE compared to the general population, one promise for the future may be whole exome sequencing, which can provide useful information about host characteristics that have the potential to influence the pathogenic mechanism of SSPE and may provide a deeper understanding of the clinical outcomes of this disease.

## 9. Conclusions

Since clinical suspicion is extremely important for establishing the SSPE diagnosis, awareness programs are necessary worldwide.

In addition, a National Registry of these patients would be extremely useful, as it may be assumed that the actual number of cases is unknown, especially since, as mentioned above, the incidence of SSPE is expected to increase over the next 5–10 years.

As long as therapeutic progress remains modest and the disease’s progression can only be slowed down without improving the prognosis, the only way to combat this severe disease is through prevention, specifically the administration of the measles vaccine.

The main objective of this study is therefore twofold: to raise public awareness of this rare disease—the recognition and diagnosis of which are essential—and to emphasize the vital importance of vaccination against measles, the only effective measure for preventing SSPE. Currently, the disease’s progression can at best be slowed, without improving the prognosis, and in most cases, it leads to severe disability and death.

## Figures and Tables

**Table 1:** Clinical stages of SSPE [26,40].

Stage	Clinical Manifestations
Stage I	Personality changes, strange behavior, poor school performance, subtle myoclonic jerks
I A	Mild mental and/or behavioral changes
I B	Marked mental changes
Stage II	Massive, repetitive, and frequent myoclonic jerks, speech deterioration, gait disturbances, pyramidal signs, seizures, and dementia
II A	Myoclonus and/or other involuntary movements and epileptic seizures
II B	Focal deficits (speech disorders, loss of vision, and limb weakness)
II C	Marked involuntary movements, severe myoclonus, or focal deficits enough to impair full daily activities
II D	Akinetic mutism, vegetative state, decerebrated, decorticated rigidity, or coma
Stage III	Severe cognitive decline, extrapyramidal symptoms, rigidity, decerebrated posturing, and progressive unresponsiveness
Stage IV	Coma, persistent vegetative state, autonomic failure, and akinetic mutism

**Table 2:** Dyken’s criteria for the diagnosis of Subacute Sclerosing Panencephalitis.

Major	Minor
Elevated CSF measles antibody titers (≥1:4) and in serum (≥1:256) Typical or atypical clinical history -Typical: acute, rapidly progressive; subacute progressive, chronic progressive, chronic relapsing–remitting -Atypical: seizures, prolonged stage I, unusual age (infancy/adult)	1. Typical EEG: periodic slow-wave complexes (“Radermecker” complexes); these discharges coincide with myoclonic jerks 2. CSF gammaglobulines (≥20% of total protein) or oligoclonal bands 3. Brain biopsy suggestive of panencephalitis, with inclusion bodies inside neurons and glial cells, neuronal loss, gliosis, and evidence of chronic viral infection 4. Molecular diagnostic test to identify the mutated genome of the measles virus

**Table 3:** Patients’ clinical stage, EEG, and MRI findings at admission and treatment.

Case	Clinical Stage at Admission	EEG	MRI
1	2A	Slow generalized background rhythm, with periodic discharges of high-amplitude delta wave complexes	T2 Flair hyperintensity in the left globus pallidus.
2	2B	Typical symmetrical periodic slow wave complexes	Extensive areas of T2 FLAIR hyperintensity, with mild T1 hypointensity located symmetrically in the deep periventricular white matter bilaterally, in the fronto-temporo-occipital subcortical white matter, and in the corpus callosum.
3	2A	Synchronous multifocal epileptiform activity structured in periodic high-amplitude wave complexes	Normal
4	2C	Slow theta-delta rhythm with repetitive sharp delta discharges.	T2 FLAIR hyperintensity in the right hippocampus, reduced anterior cortical intensity with no contrast uptake, and discrete focal hyperintensities in the bilateral thalamic white matter (demyelination).
5	2B	Slow background rhythm, with periodic high-amplitude discharges	Normal
6	2C	Diffuse slow background electric activity; generalized rhythmic synchronous discharges of slow wave complexes	Symmetrical generalized T2 FLAIR hyperintensity at the pontine level and middle cerebellar peduncles, with additional involvement in the supratentorial temporo-parieto-occipital regions and right frontal cortico-subcortical areas, in the corpus callosum and left parieto-occipital region.
7	2B	Background pattern with medium and hypovolted waves, and periodic generalized high-amplitude slow wave discharges	Diffuse symmetrical, bilateral periventricular T2 FLAIR signal into the frontal, parietal and occipital white matter and T2 hypersignal in the corpus callosum.

**Table 4:** Treatment, follow-up period, and clinical stage at the end of the study period.

Case	Treatment	Follow-Up (Months)	Actual Clinical Stage
1	Isoprinosine, levetiracetam, valproic acid, clonazepam	6	4
2	Isoprinosine, levetiracetam, valproic acid, carbamazepine	41	4
3	Isoprinosine, levetiracetam, valproic acid	37	3
4	Isoprinosine, amantadine, levetiracetam, valproic acid, clonazepam	25	4
5	Isoprinosine, levetiracetam, carbamazepine, clonazepam	17	3
6	Isoprinosine, levetiracetam, valproic acid	12	3
7	Isoprinosine, levetiracetam, valproic acid	4	Death

## Data Availability

All clinical data and materials presented in this article are available upon reasonable request from the corresponding author.

## Abbreviations

The following abbreviations are used in this manuscript:

ADHD	deficit hyperactivity disorder
CNS	central nervous system
EEG	electroencephalogram
ELISA	enzyme-linked immunosorbent assay
IFN	Interferon
IgG	immunoglobulin G
IgM	immunoglobulin M
IU	international units
MRI	Magnetic Resonance Imaging
MRS	Magnetic Resonance Spectroscopy
PCR	polymerase chain reaction
RNA	ribonucleic acid
SARS-CoV-2	severe acute respiratory syndrome coronavirus 2
SSPE	Subacute sclerosing panencephalitis
Th	T helper
UNICEF	United Nations Children’s Fund
WHO	World Health Organization
